# Cognitive and Disease-Modifying Effects of 11β-Hydroxysteroid Dehydrogenase Type 1 Inhibition in Male Tg2576 Mice, a Model of Alzheimer's Disease

**DOI:** 10.1210/en.2015-1395

**Published:** 2015-08-25

**Authors:** Karen Sooy, June Noble, Andrew McBride, Margaret Binnie, Joyce L. W. Yau, Jonathan R. Seckl, Brian R. Walker, Scott P. Webster

**Affiliations:** University/BHF Centre for Cardiovascular Science (K.S., J.N., A.M., M.B., J.L.W.Y., J.R.S., B.R.W., S.P.W.), Queen's Medical Research Institute, and Centre for Cognitive Aging and Cognitive Epidemiology (J.L.W.Y., J.R.S.), University of Edinburgh, Edinburgh EH16 4TJ, United Kingdom

## Abstract

Chronic exposure to elevated levels of glucocorticoids has been linked to age-related cognitive decline and may play a role in Alzheimer's disease. In the brain, 11β-hydroxysteroid dehydrogenase type 1 (11β-HSD1) amplifies intracellular glucocorticoid levels. We show that short-term treatment of aged, cognitively impaired C57BL/6 mice with the potent and selective 11β-HSD1 inhibitor UE2316 improves memory, including after intracerebroventricular drug administration to the central nervous system alone. In the Tg2576 mouse model of Alzheimer's disease, UE2316 treatment of mice aged 14 months for 4 weeks also decreased the number of β-amyloid (Aβ) plaques in the cerebral cortex, associated with a selective increase in local insulin-degrading enzyme (involved in Aβ breakdown and known to be glucocorticoid regulated). Chronic treatment of young Tg2576 mice with UE2316 for up to 13 months prevented cognitive decline but did not prevent Aβ plaque formation. We conclude that reducing glucocorticoid regeneration in the brain improves cognition independently of reduced Aβ plaque pathology and that 11β-HSD1 inhibitors have potential as cognitive enhancers in age-associated memory impairment and Alzheimer's dementia.

Glucocorticoids have long been recognized to impact on cognitive function, especially with aging (1–3). Older individuals who exhibit learning and memory impairments have elevated glucocorticoid levels that parallel both cognitive deficits and shrinkage of the hippocampus, a key locus for memory formation. The hippocampus expresses a high density of corticosteroid receptors, both the lower affinity glucocorticoid receptor and the higher affinity mineralocorticoid receptor, and these receptors are also abundant in other neocortical regions associated with cognition (4). Elevated glucocorticoid concentrations in vitro and in vivo promote biochemical, electrophysiological, and structural changes in hippocampal neurons, which associate with poorer memory formation (5, 6). Manipulations which maintain low glucocorticoid levels from birth (neonatal programming) or midlife (adrenalectomy and low dose steroid replacement) prevent the emergence of cognitive deficits with age (7).

Some patients with dementia, including those with Alzheimer's disease (AD), have elevated circulating cortisol levels, which may contribute to AD pathogenesis (8, 9). It has been postulated that excess glucocorticoids increase levels of amyloid precursor protein (APP) and APP cleaving enzyme (β-site APP-cleaving enzyme [BACE]), leading to increased amyloid Aβ (Aβ) formation, reduced Aβ degradation via attenuation of insulin degrading enzyme (IDE), and increased τ-expression (10). Other relevant glucocorticoid actions include hyperglycemia/insulin resistance, angiopathic and antiangiogenic actions, increased excitatory (N-methyl-D-aspartate) neurotransmission and postsynaptic calcium signaling promoting neurotoxicity, metabolic endangerment of neurons, and deleterious alterations in neuroimmune function (11).

Glucocorticoid action via intracellular mineralocorticoid receptor and glucocorticoid receptor is determined not only by circulating steroid levels but also by target tissue concentrations, modulated by intracellular metabolism by the isozymes of 11β-hydroxysteroid dehydrogenase (11β-HSD) (12). The adult forebrain expresses 11β-HSD type 1, which catalyzes conversion of inert 11-keto corticosteroids (cortisone, 11-dehydrocorticosterone) to active cortisol and corticosterone. 11β-HSD1 levels are increased in the aging rodent hippocampus and cortex and correlate with cognitive decline (13). Transgenic mice modestly overexpressing 11β-HSD1 in the forebrain show premature memory decline with aging, whereas 11β-HSD1 null mice on two distinct genetic backgrounds and even heterozygous null mice (with 50% less enzyme) resist cognitive decline with aging in a variety of tests (14). This protection associates with loss of the age-associated rise in intrahippocampal corticosterone levels but without changing plasma corticosterone levels (13).

Treatment of already aged mice with selective 11β-HSD1 inhibitors improves spatial memory performance. Effects are rapid, occurring within hours to days (15–17). Moreover, in small randomized placebo-controlled trials, the nonselective 11β-HSD inhibitor carbenoxolone improved memory in healthy aging men and in patients with type 2 diabetes (18). Whereas 11β-HSD1 inhibition improves glucose homeostasis and other metabolic parameters in obesity, metabolic changes were not correlated with cognitive effects in aged rodents or humans. These results support examination of selective 11β-HSD1 inhibitors in the treatment of age-related cognitive impairments.

Here we examined a crucial issue: whether selective 11β-HSD1 inhibition alters cognition and pathology in AD. We used a murine AD model, the well-characterized Tg2576 mouse, which bears a mutated human *APP* gene. We generated and used UE2316, a novel and selective inhibitor of both human and rodent 11β-HSD1 with a low nanomolar IC_50_ value and high penetration into the brain (19, 20).

## Materials and Methods

### Selective 11β-HSD1 inhibitor UE2316

UE2316 ([4-(2-chlorophenyl-4-fluoro-1-piperidinyl][5-(1H-pyrazol-4-yl)-3-thienyl]-methanone) was synthesized by High Force Ltd according to methods previously described (21). In vitro screening of UE2316 potency in human embryonic kidney-293 cells stably transfected with *hsd11b1* (22) showed a greater median IC_50_ than our previously reported compound UE1961 (15, 20). The inhibition of 11β-HSD1 activity in tissue extracts was quantified as previously described (22). Liver brain and white adipose tissues were collected and snap frozen on dry ice. Frozen tissue (50–80 mg) was homogenized in 700 μL of chilled Krebs buffer and a cleared homogenate prepared by centrifugation at 3500 rpm for 5 minutes. The protein concentration of this homogenate was determined by a Bradford assay. For the assay, 25 μL of 10 mM nicotinamide adenine dinucleotide phosphate was added to 250 μg of the homogenate in a final volume of 200 μL chilled Krebs buffer and incubated at 37°C for 20 minutes. 3H-cortisone (25 μL of 200 nM) was then added and the assay incubated for a further 15 minutes prior to termination by rapid freezing on dry ice. ^3^H-cortisone to ^3^H-cortisol conversion was determined in 50 μL of the defrosted reaction by capturing liberated ^3^H-cortisol on anticortisol (HyTest Ltd)-coated scintillation proximity assay beads (protein A-coated YSi; GE Healthcare). The percentage inhibition was determined by measuring the conversion of ^3^H-cortisone to ^3^H-cortisol relative to that in tissue from vehicle treated mice.

### Animals

All in vivo experiments were performed under a project license issued under the UK Scientific Procedures (Animals) Act, 1986, and with local ethical committee approval. Male C57BL/6 mice were obtained from Harlan. Male mice were chosen to eliminate the potential effects of gonadal hormonal fluctuations observed in females. Animals were group housed under controlled lighting (lights on from 7:00 am to 7:00 pm) and temperature (22°C), with access to food and water ad libitum. The experimental procedures are summarized in Table 1.

**Table 1.** Treatment Table

Strain	Age at t = 0	Dose	Route	Test	Postdose Test	n per Group
C57BL/6	8–10 wk	10 mg/kg	Oral	Tissue inhibition	Day 1	3 (vehicle), 3 (drug)
C57BL/6	8–10 wk	10 mg/kg	sc	Tissue inhibition	Day 1	3 (vehicle), 3 (drug)
C57BL/6	24 mo	100 ng/h	icv	Tissue inhibition	Day 9	9 (vehicle), 8 (drug)
				Y maze	Day 8	
C57BL/6	22 mo	5 mg/kg/d	sc	Y maze	Day 10	6 (vehicle), 8 (drug)
				Passive avoidance	Days 13 and 14	
		15 mg/k/d		Y maze	Day 10	
				Passive avoidance	Days 13 and 14	
Tg2576	14 mo	10 mg/kg/d	sc	Morris water maze	Day 14	10 (vehicle), 10 (drug)
				Open field	Day 23	
				Spontaneous alternation	Day 26	
				Passive avoidance	Days 27 and 28	
				Immunohistochemistry	Day 29	
B6;SJL	14 mo	10 mg/kg/d	sc	Morris water maze	Day 14	10 (vehicle), 10 (drug)
				Open field	Day 23	
				Spontaneous alternation	Day 26	
				Passive avoidance	Days 27 and 28	
Tg2576	6–7 mo	30 mg/kg/d	Oral	Passive avoidance	Weeks 15 and 41	13 (vehicle), 23 (drug)
				Open field	Week 38	
				Spontaneous alternation	Week 39	
				Immunohistochemistry	Week 44	5 (vehicle), 5 (drug)
				Morris water maze	Week 52	6 (vehicle), 9 (drug)
BL6;SJL	6–7 mo	30 mg/kg/d	Oral	Open field	Week 38	5 (vehicle), 5 (drug)

For measurement of pharmacodynamic inhibition after oral administration, oral gavages with vehicle (38% polyethylene glycol [PEG], 2% dimethyl sulfoxide [DMSO], 60% saline; Sigma) or UE2316 dissolved in vehicle were performed in the morning in animals aged 8–10 weeks (n = 3 per dose). Animals were killed after the dosing (1, 4, and 6 h) and the tissues retained for analysis of 11β-HSD1 inhibition.

For assessment of pharmacodynamic inhibition after the sc administration, C57BL/6 mice (8–10 mo; Harlan UK) (n = 3 per group) were treated with either vehicle (50:50 DMSO-PEG; Sigma) or 10 mg/kg/d UE2316 in vehicle via sc implanted Alzet osmotic minipumps (model 2004; Charles River) for 14 days. Animals were killed at this stage and the tissues retained for analysis of 11β-HSD1 inhibition.

To show that the effects of UE2316 were not merely due to any peripheral metabolic actions of 11β-HSD1 inhibition, the agent was administered intracerebroventricularly to aged male C57BL/6 mice (24 mo, obtained from an in-house stock) were treated with either vehicle (artificial cerebrospinal fluid [CSF]; Alzet; Charles River) (n = 9) or 100 ng/h UE2316 in artificial CSF (n = 8) administered via intracerebroventricular (icv) infusion, for 9 days, as previously described (23).

For assessment of the effects of UE2316 on cognition in aging animals, aged male C57BL/6 mice (22 mo; Harlan UK) were treated with either vehicle (n = 6), 5 mg/kg/d UE2316 (n = 8) or 15 mg/kg/d UE2316 (n = 8) in vehicle (50:50 DMSO-PEG; Sigma) by sc implanted Alzet osmotic minipumps (model 2004; Charles River) for 23 days, with body weights monitored at the start and end of the treatment.

For the assessment of the effects of UE2316 in a model of AD, male Tg2576 (24) and age-matched genetic control (BL6;SJL) littermates were obtained from Taconic Europe. Animals were singly housed due to aggressive behavior. In the short-term treatment study, 14-month-old mice of each genotype (n = 10 per group) were allocated at random to receive either UE2316 (10 mg/kg/d) or vehicle (50:50 DMSO-PEG; Sigma) via two Alzet osmotic minipumps (model 2004) implanted sc to provide sufficient volume of drug or vehicle for 29 days. Food intake and body weight were monitored weekly throughout. For the long-term study in which UE2316 was administered by incorporation in the diet, 6- to 7-month-old Tg2576 male mice that were screened for eye color, coat color, and rd1 homozygosity for the *pde6b*^rd1^ retinal degeneration mutation by Taconic were fed with control diet (RM1) (n = 16) or with RM1 containing 175 ppm UE2316 (for a calculated dosage of 30 mg/kg/d) (n = 32) (Special Diet Services) for up to 57 weeks. Food intake and body weights were monitored weekly throughout the experiment, and drug dosages were calculated based on the average daily food intake. Each cohort of mice underwent repeated longitudinal cognitive testing.

### Behavior

Mice were acclimatized to the behavior room for at least 1 hour before all procedures to minimize stress. All behavioral testing was conducted during the day between 9:00 am and 12:00 pm.

### Memory in passive avoidance

Passive avoidance was assessed over 2 days (for aging studies in C57BL/6 mice on days 13 and 14 after the start of treatment for the short-term UE2316 study in Tg2576 mice on days 27 and 28 and at weeks 15 and 41 in the long-term UE2316 Tg2576 study) in a step-through light/dark box passive avoidance apparatus (Ugo Basile Comerio) (13). On the first day, the latency to enter the dark compartment from the light compartment was measured, with the door to the dark compartment opening 30 seconds after the start of the trial. Twenty-four hours later, the latency to enter the dark compartment was repeated, which was followed by a mild 0.3-mA, 2-second foot shock in the dark compartment. The mice were then retested 6 hours later for the latency to enter the dark compartment, this time without a foot shock. The latencies were measured automatically by the device after the opening of the door separating the light and dark compartments with a maximal time allowed of 300 seconds. Mice that did not enter the dark compartment were eliminated from analysis.

### Y maze testing

Y maze testing of spatial hippocampal memory was performed as previously described with a 2 -hour intertrial interval (ITI) on day 10 after the start of treatment in the aged UE2316 study (15). For the UE2316 icv administration study, the Y maze testing was performed on day 8 of treatment. The amount of time spent in each arm was measured and analyzed using AnyMaze software (Stoelting).

### Open field testing

The open field test was performed on day 23 of the short-term Tg2576 study and week 38 of the long-term Tg2576 study. Mice were placed in an open field box (60 × 60 cm) marked off into 16 equal squares. The outer row of squares adjacent to the walls of the box are considered less anxiogenic than the inner squares. For a 5-minute period, the number of crossings, time, and distance (movement of all four legs into a new square) into each square was noted. Total movement in the maze reflects general activity and the relative movement in the inner zone is correlated to the anxiety state of the mouse.

### Spontaneous alternation

Spontaneous alternation, a test of working hippocampal memory, was tested after 26 days of treatment in the short-term-treated Tg2576 mice and after 39 weeks of treatment in the long-term diet study. Mice were placed in a Y maze apparatus consisting of three enclosed black Plexiglas arms (50 cm long, 11 cm wide, and 10 cm high), with prominent extramaze visual cues. Mice were allowed to explore the maze for 5 minutes after starting in a randomly chosen start arm. The order and number of arm entries by each mouse in the 5-minute test period were recorded. Percentage spontaneous alternation was calculated using the following formula: percentage spontaneous alternation = (number of alternations [which is entries into three different arms consecutively]/[number of arms entered minus 2]) × 100.

### Morris water maze

Morris water maze testing was performed as previously described on day 14 of the short-term study and week 52 of the long-term study (25). For the short-term treatment study in Tg2576 mice, mice that did not swim (ie, did not engage with the task) during the visible platform test, in which a visual cue (ie, stacked Lego blocks) was placed on the submerged platform in the tank and no visuospatial clues were present (curtains were closed) were eliminated from analysis. The mice undertook four trials per day with a 20-minute ITI and a maximum swim time of 90 seconds per trial for 4 days. Latency, swim speed, and the percentage of time spent in each quadrant of the pool were measured by Watermaze software (Actimetrics). In the long-term treatment study, mice were initially assessed after 12 months of treatment for their ability to engage in the visible platform test. In this instance, mice that were able to find the platform and improve their latencies after 2 days of 4 × 90-second trials were then tested in the spatial water maze, in which the platform remained submerged without a visual cue on top and the mice used spatial clues located around the maze (curtains open). Mice were tested in 4 × 90-second trials per day over 6 days. Twenty-four hours after the final spatial water maze trial, the mice were then tested in the 90-second probe test, in which the hidden platform was removed from the tank and the percentage of time spent swimming in the target quadrant was measured.

After behavioral testing, the mice were killed by cervical dislocation on day 29 of vehicle or UE2316 treatment in the short-term study or after 44 and 57 weeks in the long-term study. The brains were removed and hemisected coronally. Half of the brain was dissected and cortex, hippocampus and cerebellum were immediately frozen on dry ice and stored at −80°C for further analysis. The other half was fixed in 4% paraformaldehyde in PBS (VWR) and cryoprotected in 30% sucrose (Sigma) overnight at 4°C before storage at −80°C for immunohistochemistry.

### Immunohistochemistry

All immunohistochemistry was performed on free-floating, 25-μm sections stored at −20°C in cryoprotectant (50 mM phosphate buffer, 25% glycerol, 25% ethylene glycol; Sigma). Sections were transferred to a 12-well tissue culture plate with Netwell inserts (VWR) and washed in PBS. Antigen retrieval was performed by heating the sections in sodium citrate buffer (pH 6.0) (Sigma) at 95°C for 15 minutes, followed by peroxidase treatment (1% H_2_O_2_ in PBS; Sigma) for 30 minutes to remove endogenous peroxidase activity, washed, and then blocked with the appropriate serum for 1 hour followed by overnight incubation at 4°C with the antibody of choice. For staining using the 6E10 antibody for visualization of amyloid β-plaques, the sections were blocked using the mouse-on-mouse Ig blocking reagent (Vector Laboratories) followed by overnight incubation with a 1:1000 dilution of Aβ 1–16 mouse monoclonal antibody (6E10) (Covance, Cambridge Bioscience). After washing, sections were incubated with a secondary antibody for 1 hour at room temperature. Staining was visualized using the Vectastain avidin biotin complex kit and diaminobenzidine peroxidase substrate kit (Vector Laboratories). The sections were then mounted on Superfrost Plus slides (VWR), dehydrated, and coverslipped. The number of 6E10-positive plaques per brain area was counted by an experimenter blinded to the treatment group using a Zeiss Axioskop and the KS300 imaging program (Zeiss). The plaque area, measured using the same program, was expressed as plaque area divided by the total area of the brain region. Iba-1 antibody was purchased from Abcam. Goat and rabbit serums were purchased from Sigma. Biotinylated rabbit antigoat IgG antibody and biotinylated rabbit antisheep IgG antibody were purchased from Vector Laboratories.

### Western blotting

Protein extracts were prepared from the brain areas by homogenization in Krebs buffer containing protease inhibitor (Roche) followed by centrifugation at 3000 rpm for 5 minutes. Protein concentration of the supernatant was measured using the Bradford assay (Bio-Rad Laboratories). Proteins were separated by SDS-PAGE using NuPAGE Novex 4%–12% bis-Tris gels (Invitrogen) and transferred to nitrocellulose membranes (0.2 μm pore size; Invitrogen). Membranes were blocked for 1 hour at room temperature in 5% nonfat dry milk blotting grade blocker (Bio-Rad Laboratories) in PBS (pH 7.4) containing 0.1% Tween 20 and then incubated overnight with shaking at 4°C with the primary antibody diluted in blocking reagent. This was followed by incubation at room temperature in the appropriate secondary antibody. IDE, PSD95, A disintegrin and metalloproteinase domain-containing protein 10 (ADAM10), synaptophysin, and CD31 antibodies were purchased from Abcam. The anti-BACE 1 N terminus (46–62) antibody was sourced from Sigma. Mouse β-tubulin antibody was purchased from Merck-Millipore. Goat-antirabbit IgG antibody was obtained from Licor Biosciences UK. Alexa Fluor 680 donkey antisheep IgG (H+L) and Alexa Fluor 680 rabbit antigoat IgG antibodies were purchased from Invitrogen. Proteins were visualized and band intensities were quantified using the Odyssey infrared imaging system (LiCor Biosciences UK).

### Statistical analysis

Data are expressed as mean ± SEM. Groups were compared by an ANOVA. When the ANOVA was significant, post hoc tests were performed as indicated in the figure legends. Differences were considered significant at a value of *P* < .05.

## Results

### UE2316 inhibits 11β-HSD1 in the brain

**Figure 1.**
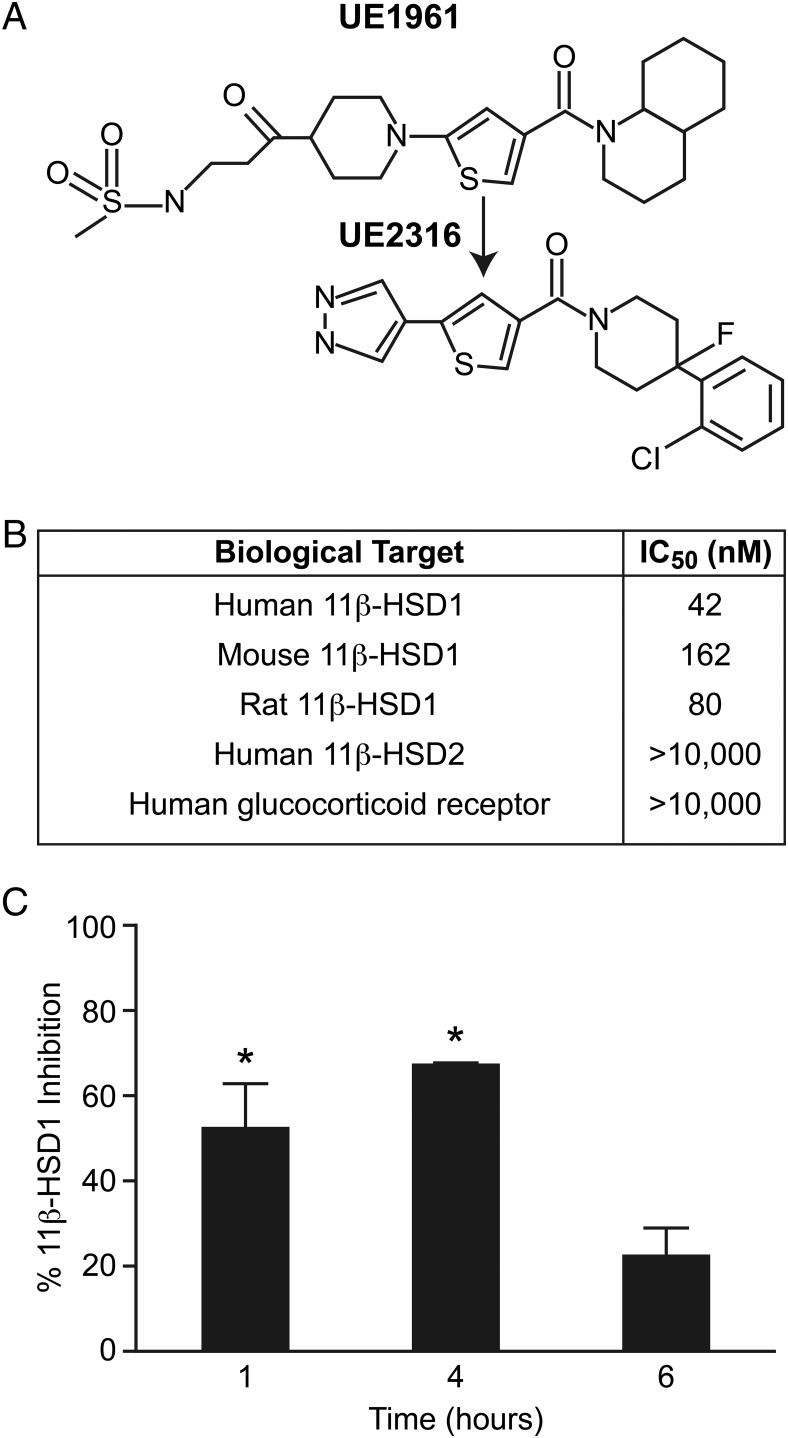
UE2316 Characteristics. A, Structural comparison of UE1961 and UE2316. B, Potency and selectivity of UE2316. C, Male C57BL/6 mice were treated with a single 10-mg/kg oral dose of UE2316 (n = 3 animals per time point) and inhibition by scintillation proximity assay was assessed 1, 4, and 6 hours after dosing and expressed as the percentage inhibition compared with values obtained in vehicle-treated mice (one-way ANOVA, *P* = .009; Bonferroni's post hoc comparisons). *, *P* < .05 vs vehicle.

We previously reported the discovery and pharmacological effects of the selective 11β-HSD1 inhibitor UE1961, which was based on a thiophene amide scaffold (15). However, this molecule has suboptimal potency and pharmacokinetic properties for progression to late-stage preclinical development. Further medicinal chemistry optimization of this compound, replacing the decahydroquinoline and substituted piperidine groups flanking the thiophene core, led to the identification of UE2316 (Figure 1A). UE2316 displays greater potency than UE1961, excellent selectivity, and an improved drug metabolism and pharmacokinetic profile for use in in vivo studies (Figure 1B). Pharmacodynamic inhibition of 11β-HSD1 in tissues was confirmed after administration by oral and sc routes. Single-dose oral administration of UE2316 to C57BL/6 mice induced significant ex vivo inhibition of 11β-HSD1 in the brain for at least 4 hours (Figure 1C), whereas constant infusion of 10 mg/kg/d of UE2316 over 14 days by sc Alzet osmotic minipumps also produced 34.2% ± 8.3% inhibition of 11β-HSD1 in the brain (data not shown). The results from these studies were in agreement with those from previous studies (19, 20). UE2316 was thus chosen to investigate the effects of chronic 11β-HSD1 inhibition on cognitive impairment and AD pathology in mouse models using either sc or oral administration.

### UE2316 acts in the brain to improve cognition in aged wild-type mice

**Figure 2.**
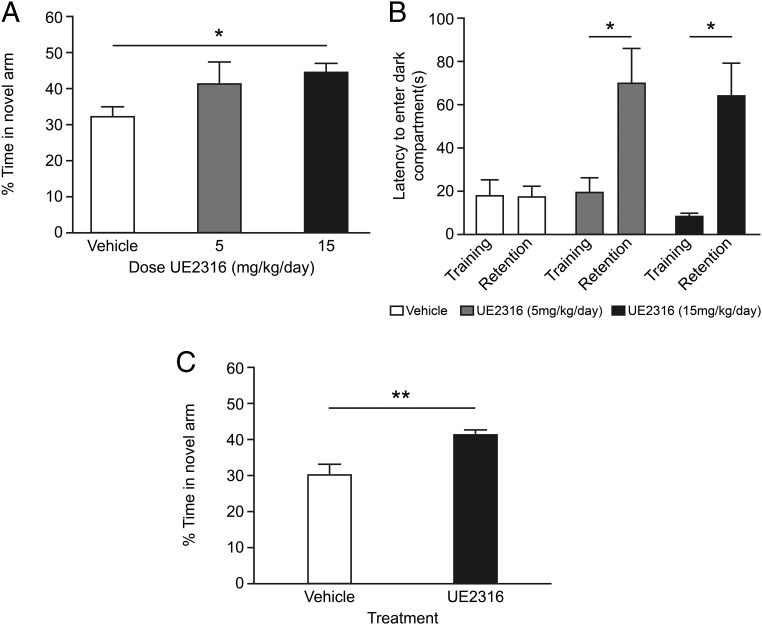
UE2316 improved spatial and fear-associated memory in aged C57BL/6 mice. Aged 22-month-old C57BL/6 mice were treated with 0 (n = 6), 5 (n = 8), or 15 (n = 8) mg/kg · d UE2316 for 23 days via sc implanted osmotic minipumps. A, Spatial memory was assessed by the Y maze on day 10 of treatment. The initial 1 minute ITI was performed prior to surgery. Treatment with 15 mg/kg · d UE2316 increased the time spent in the novel arm in the 2-hour ITI compared with vehicle-treated animals (*, *P* < .05 by a Student's *t* test with Bonferroni correction), and a trend for improvement was seen with the 5-mg/kg · d dose. B, Passive avoidance was analyzed on days 13 and 14 of treatment. Both 5 mg/kg · d (*, *P* = .02 vs vehicle by Student's *t* test) and 15 mg/kg · d (*, *P* = .03 by Student's *t* test) UE2316 improved latency in the retention trial compared with the vehicle-treated group (by two way repeated measures ANOVA drug interaction with training vs retention, *P* < .05). C, Similarly aged C57BL/6 mice were treated with an infusion of either vehicle (artificial CSF) (n = 9) or 100 ng/h UE2316 (n = 8) via icv cannulas. Spatial memory was assessed by the Y maze on day 9 of treatment. The initial 1-minute ITI was performed prior to surgery. Treatment with UE2316 increased the time spent exploring the novel arm during the 2-hour ITI compared with the vehicle-treated controls (**, *P* = .005 by Student's *t* test).

To assess the effects of UE2316 on memory in cognitively impaired mice, C57BL/6 male mice aged 22 months were randomly assigned to treatment with 0, 5, or 15 mg/kg/d of UE2316 via sc implanted Alzet minipumps for 14 days. In the Y maze, a nonstressful test of hippocampal-associated spatial memory, there was a significant increase in time spent exploring the novel arm after a 120-minute ITI in mice receiving 15 mg/kg/d UE2316 compared with vehicle-treated controls (Figure 2A). Cognition was also assessed in the passive avoidance task, which tests emotional and fear associated memories (13). During the retention phase of the passive avoidance test, UE2316 increased latency to enter the dark compartment at both 5 and 15 mg/kg · d compared with vehicle-treated mice, indicating improved memory (of the foot shock) (Figure 2B).

To investigate whether brain-specific inhibition of 11β-HSD1 was responsible for these improvements in cognition, we delivered UE2316 directly to the brain of aged (24 mo old) C57BL/6 mice at an appropriate concentration via icv administration for 9 days. Postmortem analysis of whole-brain samples revealed 39.9% ± 5.5% inhibition of 11β-HSD1 was achieved with a 100 ng/h infusion. Vehicle-treated aged controls showed impaired spatial memory in the Y maze (similar times spent in all three arms) as previously reported (15). Mice treated with icv UE2316 spent more time exploring the novel arm of the Y maze than vehicle-treated mice, indicating an improvement in spatial memory after 8 days of treatment (Figure 2C).

### UE2316 improves cognition in a murine model of AD

After the confirmation of the effects of 11β-HSD1 inhibition using UE2316 in age-related cognitive impairment, we examined effects of short-term UE2316 administration in the Tg2576 mouse model of AD. Tg2576 mice carry a transgene with mutations at amino acids 670 and 671 in the human *APP* gene under the control of the hamster prion promoter, which leads to the accumulation of Aβ plaques in the brain from 9 to 12 months of age with consequent cognitive impairment (24). Singly housed age-matched male mice were separated into four groups of 10 mice per group: wild-type or Tg2576 mice administered vehicle or UE2316. Mice were treated by two sc implanted Alzet minipumps from 14 months of age for 29 days. No treatment-related adverse effects on final body weight, daily food intake, or adrenal size were observed. However, as previously reported, Tg2576 mice weighed less than their wild-type littermates throughout the study despite consuming more food (Supplemental Figure 1, A and B), which may reflect their increase in locomotor activity and hypothalamic dysfunction (26, 27).

**Figure 3.**
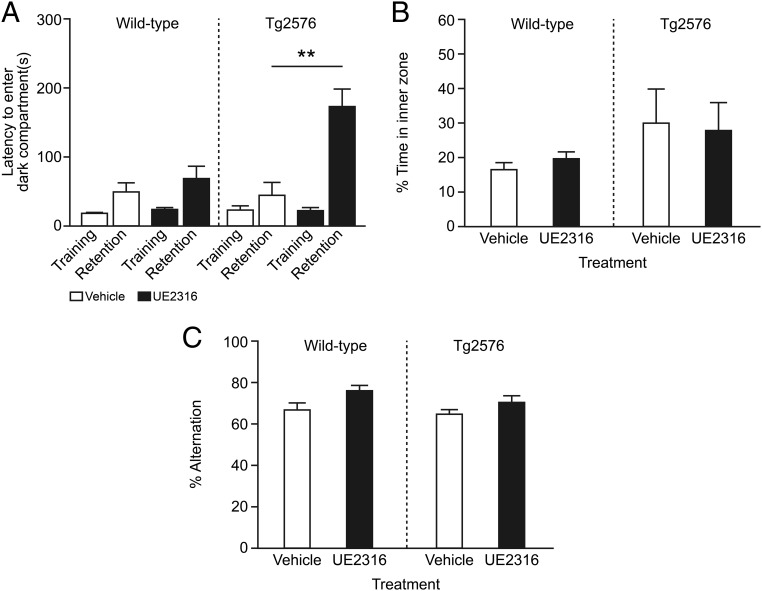
UE2316 improved fear-associated behavior in the passive avoidance test in Tg2576 mice. Tg2576 and wild-type mice (n = 10 per group) were treated with vehicle or UE2316 at 10 mg/kg · d by sc Alzet minipump infusion from the age of 14 months for 29 days. A, Tg2576 mice were assessed in the passive avoidance task on days 27 and 28 of drug treatment. The latency to enter the dark compartment was assessed in the training trial (pre) and the retention trial (post). UE2316 increased latency to enter the dark compartment 6 hours after shock (two way repeated measures ANOVA drug effect, *P* < .04; **, *P* < .01 by Student's *t* test) to a greater extent in Tg2576 mice (by an increment of 171.6 ± 28.0 sec compared with 42.6 ± 21.5 sec in vehicle treated mice; **, *P* = .004 by Student's *t* test). B, Open field was performed after 23 days of treatment in vehicle- and UE2316-treated wild-type and Tg2576 mice. The percentage of time during the 5-minute trial that was spent in the inner zone of the open field apparatus was measured. There was no significant effect of treatment or genotype. C, Spontaneous alternation was assessed at day 26 of treatment. There was a trend for increased alternation with UE2316 treatment (two way ANOVA; treatment: *P* = .06).

Tg2576 mice perform poorly in the Y-maze spatial memory test due to retinal degeneration; therefore, fear-associated memory was assessed in the passive avoidance task, which is not dependent on visual acuity. In this test, performed on days 27 and 28 of drug or vehicle administration, UE2316 treatment increased latencies in reentry to the dark compartment at 6 hours after the foots hock in both control and Tg2576 mice (Figure 3A), suggesting an improvement in fear-associated memory with drug treatment. The effect of UE2316 treatment was particularly pronounced in Tg2576 mice, which may reflect a difference in sensitivity to the electrical shock in this mouse strain. In a separate open field test, no difference was observed in the time spent in the inner zone in either wild-type or Tg2576 mice with or without drug (two way ANOVA, treatment effect: *P* = .98), suggesting that UE2316 does not affect anxiety (Figure 3B). No difference in speed was observed between either strain, in the presence or absence of drug (two way ANOVA, treatment effect: *P* = .94, data not shown).

In spontaneous alternation, a test of working hippocampal memory, Tg2576 mice tended to enter more arms than the control mice (*P* = .06), which again may be due to their increased locomotion (data not shown) (26). Treatment with UE2316 led to a trend in increased percentage alternation in both wild-type and Tg2576 mice compared with vehicle (two way ANOVA, treatment effect: *P* = .06) (Figure 3C).

### UE2316 prevents cognitive decline in a murine model of AD

Effects of long-term UE2316 treatment were examined by administering UE2316 in the diet to 6- to 7-month-old Tg2576 mice over a period of 57 weeks. The mice on the UE2316-supplemented diet maintained an average daily dose of approximately 30 mg/kg/d throughout the experiment and did not exhibit any adverse effects (Supplemental Figure 2A). UE2316-treated mice tended to weigh less than vehicle-treated mice up to 44 weeks (ANOVA, *P* < .01) despite eating more food (ANOVA, *P* < .001) (Supplemental Figure 2, B and C). In contrast to the short-term study, mice were prescreened for retinal degeneration and only those that were retinal degeneration negative were included. Memory was assessed at intervals using passive avoidance, spontaneous alternation, and Morris water maze tests.

**Figure 4.**
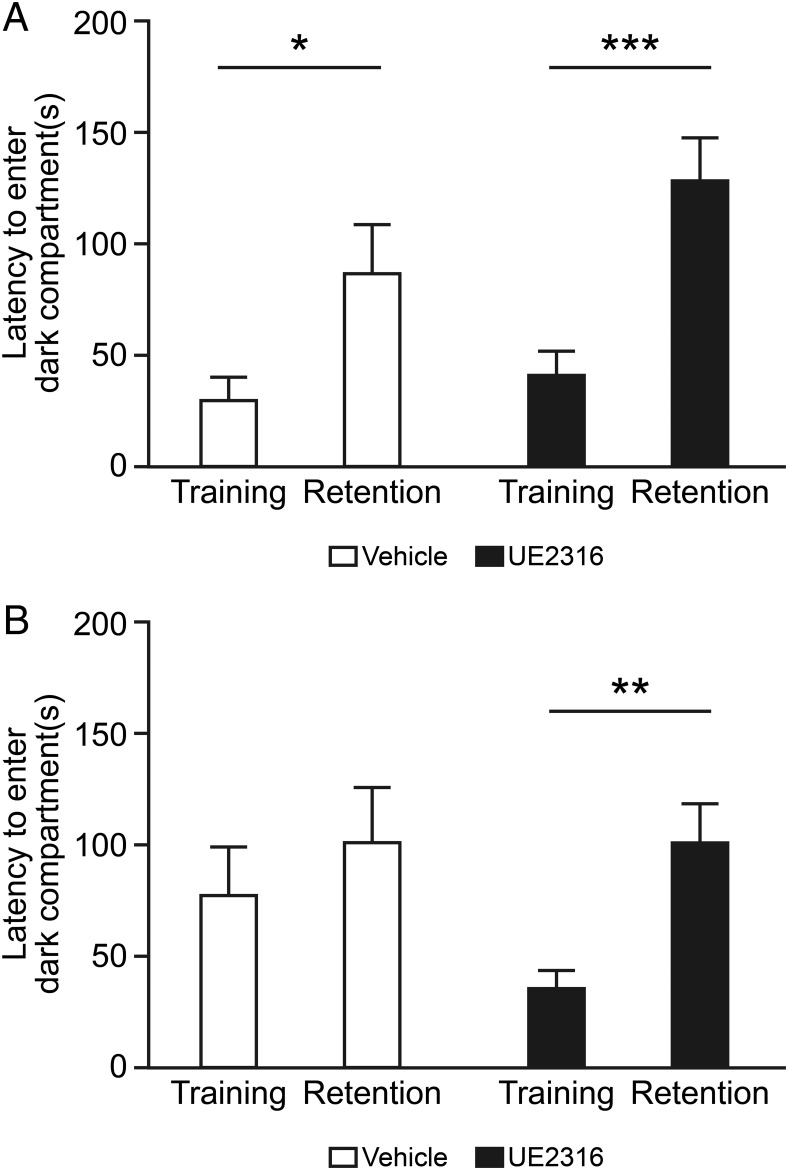
Long-term administration of UE2316 improves cognition in Tg2576 mice. Tg2576 mice were treated with either control diet (RM1, n = 16) or RM1 supplemented with 175 ppm UE2316 for an estimated dose of 30 mg/kg · d (n = 32) from the age of 6–7 months for 57 weeks. A, Passive avoidance was analyzed after 15 weeks of treatment with vehicle (n = 13) or UE2316 (n = 23). Both groups exhibited significantly increased latency to enter the dark compartment in the retention test, indicating preserved cognitive function, but there was no effect of UE2316 (by two way ANOVA training vs the retention effect, *P* < .001; drug effect, *P* = .26; by Student's *t* test; *, *P* = .03, ***, *P* = .0007). B, Passive avoidance was retested after 41 weeks of treatment with vehicle (n = 13) or UE2316 (n = 23) in chow. UE2316- but not vehicle-treated mice demonstrated a significant increase in latency to enter the dark compartment in the retention test (by Student's *t* test; **, *P* = .004).

**Figure 5.**
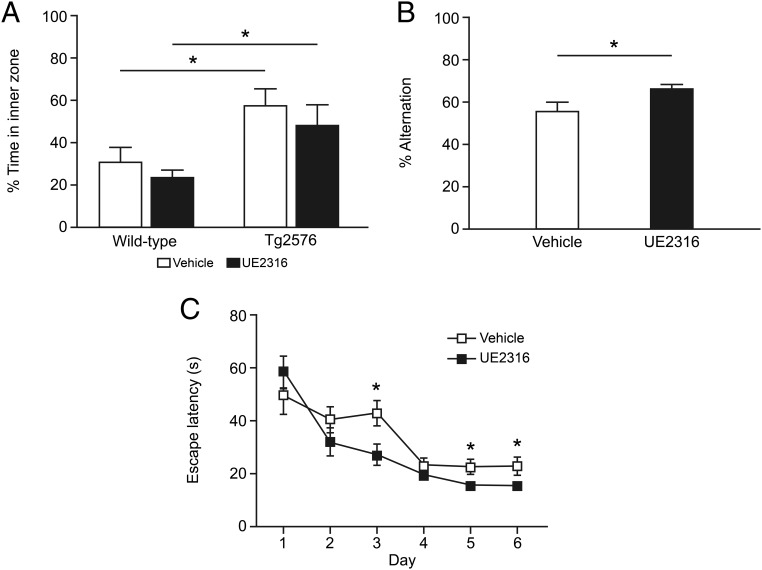
Behavior of Tg2576 mice after long-term administration of UE2316. A, Open field was performed in control (vehicle: n = 5; UE2316: n = 5) and Tg2576 mice (vehicle: n = 13; UE2316: n = 23) after 38 weeks of treatment with either normal chow or diet containing UE2316. The Tg2576 mice spent more time in the inner zone than their wild-type counterparts; however, there was no effect of drug administration (by two way ANOVA, *, *P* < .05). B, Spontaneous alternation was assessed in Tg2576 mice after 39 weeks of vehicle (n = 13) or UE2316 treatment (n = 23). UE2316-treated mice exhibited a significant increase in percentage alternation compared with control mice (by Student's *t* test; *, *P* = .04). C, The spatial Morris water maze test was performed after 52 weeks of treatment. Mice that were able to find the platform in a visible platform test were tested in their ability to find the submerged platform using spatial cues located around the testing room. UE2316-treated mice (n = 9) exhibited significant decreases in latency to find the hidden platform across the testing period compared with the vehicle-treated animals (n = 6) (two way repeated measures ANOVA; drug effect, *P* = .02, and interaction of drug with time, *P* < .01; by Student's *t* tests; *, *P* < .05 at d 3, 5, and 6).

Fear-associated memory was assessed using the passive avoidance test after 15 and 41 weeks of treatment. As expected, at 15 weeks, when mice were aged 10–11 months, similarly increased latencies were observed after training in both the vehicle- and UE2316-treated groups, consistent with preserved cognitive function at this age (Figure 4A). In contrast, after 41 weeks of treatment, when the mice were aged 16–17 months, vehicle-treated mice exhibited cognitive impairment as demonstrated by lack of prolongation of latency after training, but Tg2576 mice treated with UE2316 maintained an increase in latency to enter the dark compartment 6 hours after shock, indicating that UE2316 prevents an age-associated decline in fear-associated memory (Figure 4B). There was an increase in latency for training in vehicle-treated mice, suggesting an impairment in their ability to effectively to engage with the task. No difference in anxiety was observed with UE2316 treatment in a separate open field test (two way ANOVA, treatment effect: *P* = .25), but Tg2576 mice were less anxious compared with wild-type mice (two way ANOVA, genotype effect: *P* < .01) (Figure 5A). There was also an increase in locomotion in Tg2576 mice when compared with wild-type mice (two way ANOVA, genotype effect: *P* < .01) but no effect of treatment (two way ANOVA, treatment effect: *P* = .69).

Working memory was assessed at 39 weeks of treatment using the spontaneous alternation test. UE2316 increased spontaneous alternation (Figure 5B), although there was no difference in the total number of arm entries (data not shown).

Spatial memory was assessed in the Morris water maze after 52 weeks of treatment (mice aged 18–19 mo). Swim speeds were not affected by UE2316 treatment and there was no difference between strains (data not shown). UE2316 reduced the time taken to find the hidden platform across the testing period (Figure 5C) and increased time spent in the target quadrant during the probe test performed 24 hours after the final trial (vehicle: 34.2% ± 3.2%, UE2316: 52.1% ± 5.1%, *P* = .02), consistent with improved spatial learning and retention (Supplemental Figure 3A).

### Effect of UE2316 on Aβ plaques in the brain

Immunohistochemistry with the 6E10 antibody (28), which detects Aβ1–16, was performed on Tg2576 brains to determine whether UE2316 affected the number and volume of Aβ plaques (wild type control brains were not analyzed because these mice do not develop amyloid plaques) (see Figure 7A).

**Figure 6.**
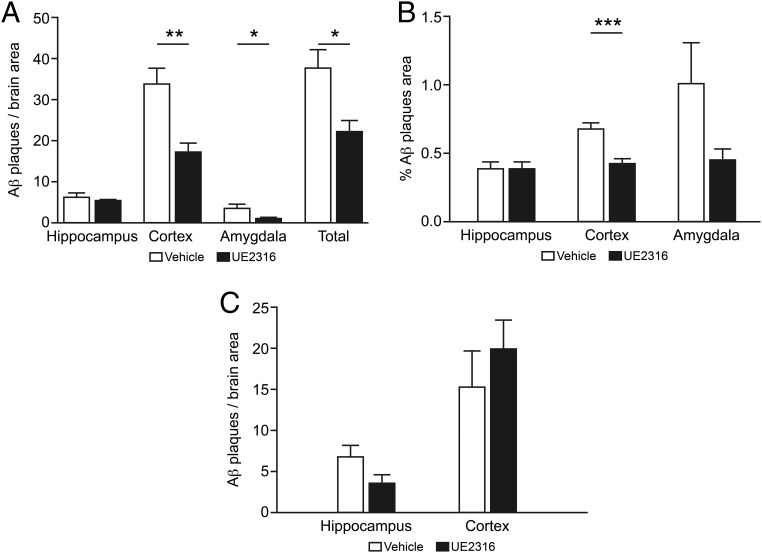
Effect of short- and long-term UE2316 administration on amyloid plaque burden in Tg2576 mice. A, Short-term plaque number. The 6E10-positive amyloid plaques were counted in at least five nonsequential sections per mouse treated for 29 days with vehicle or UE2316 (n = 10 per treatment) via sc minipumps using the KS300 imaging program, and the total number of positive plaques was expressed per area of the brain. UE2316 had no effect on plaque number in the hippocampus but decreased 6E10 staining in the cortices (Student's *t* test, **, *P* = .002), amygdala (Student's *t* test, *, *P* = .05), and whole brain (Student's *t* test, *, *P* = .01) in comparison with vehicle. B, Short term plaque area. Total plaque area of short-term (29) treated mice was measured using the KS300 imaging program and was expressed as plaque area divided by the total area of the brain region in question. The total plaque area in the cortex was decreased by UE2316 in comparison with vehicle (Student's *t* test, ***, *P* = .0001). C, Long-term plaque number. The 6E10-positive amyloid plaques were counted in at least five nonsequential sections per mouse treated for 44 weeks with vehicle or UE2316 (n = 5 per treatment) in the diet using the KS300 imaging program and the total number of positive plaques was expressed per area of the brain. UE2316 had no statistically significant effect on plaque number in the hippocampus or cortex in comparison with vehicle.

Short-term, 4-week UE2316 treatment decreased the Aβ plaque number in the cortex and amygdala but not the hippocampus of 15-month-old Tg2576 mice (Figure 6A). The total plaque number in the brains of Tg2576 mice was 54% lower in UE2316-treated than vehicle-treated mice. Plaque areas were correspondingly reduced (Figure 6B).

After chronic 44-week treatment with UE2316 (mice aged 17–18 mo), there were a similar number of Aβ plaques in the cortex and hippocampus as in mice treated with UE2316 for only 4 weeks (Figure 6C). However, there were fewer Aβ plaques in the brains of the vehicle-treated group from the 44-week study than in the vehicle-treated animals from the 4-week study. In the 44-week study, there was no significant effect of UE2316 on the number of Aβ plaques.

**Figure 7.**
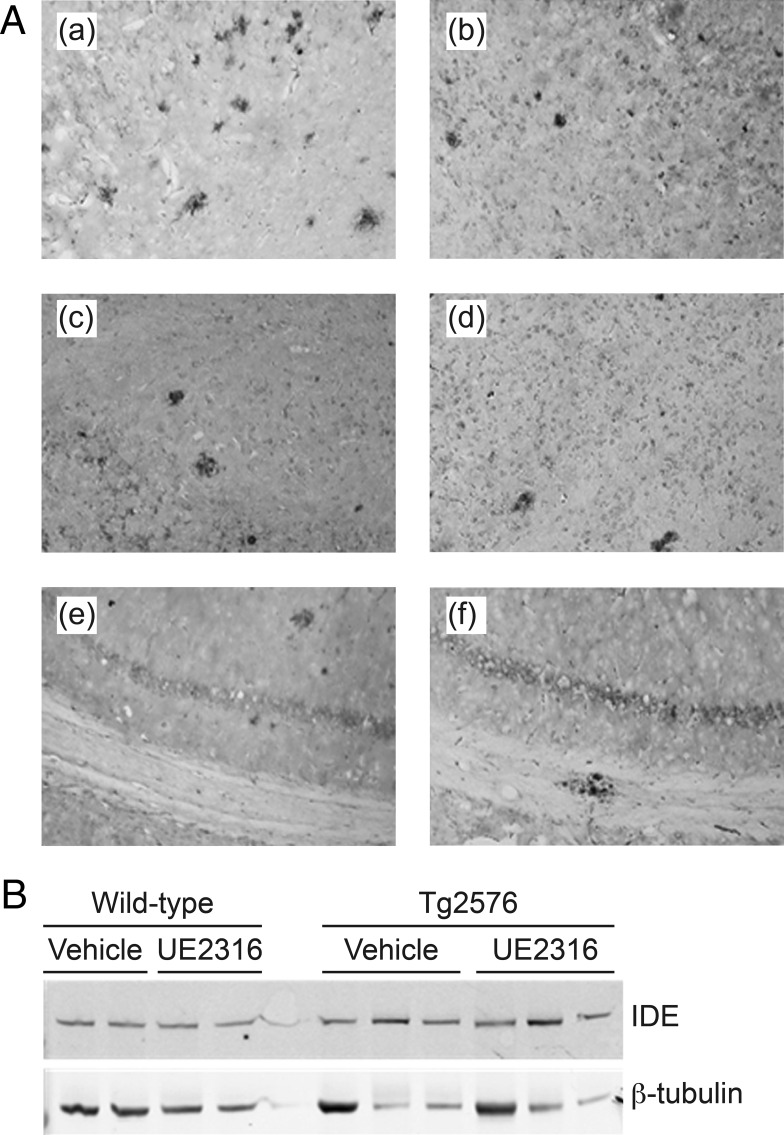
Effects on brain pathology in Tg2576 mice treated with UE2316. A, Representative brain sections from Tg2576 mouse showing amyloid plaques in brain regions stained with 6E10 antibody. a, cortex, vehicle treated; b, cortex, UE2316 treated; c, amygdala, vehicle treated; d, amygdala, UE2316 treated; e, hippocampus, vehicle treated; f, hippocampus, UE2316 treated. B, Representative Western blot of cortex protein (30 μg/sample) from 29-day treated mice. Quantitation was performed using the Odyssey Infrared imaging system and adjusted for β-tubulin. IDE levels were increased in UE2316-treated cortices compared with vehicle-treated tissues in both wild-type and Tg2576 animals (see Table 1).

To explore possible mechanisms mediating the effects of short-term UE2316 treatment on Aβ plaque burden in the cortex, we conducted Western blot analyses of selected proteins involved in Aβ generation and metabolism, the expression of which are known to be regulated by glucocorticoids (Figure 7B). UE2316 treatment did not modulate BACE protein expression in either wild-type or Tg2576 mice (Table 2). Nor did UE2316 alter ADAM10, a metalloproteinase possessing α-secretase activity involved in the nonamyloidogenic pathway of APP processing (29) (Table 2). However, UE2316 significantly increased, by 31% and 34%, respectively, IDE protein expression in the cortex of both control and Tg2576 mice (Table 2). No difference in IDE expression was found in the brains from mice treated with UE2316 for 44 weeks (IDE to β-tubulin ratio: vehicle: 0.016 ± 0.004 vs UE2316: 0.011 ± 0.002; Student's *t* test, *P* = .22).

**Table 2.** UE2316 Increased IDE Protein Levels in the Cortex of Control and Tg2576 Mice

Target	Ratio vs β-Tubulin				*P* Value: Two-Way ANOVA of Effect of UE2316
	Wild-Type Vehicle	Wild-Type UE2316	Tg2576 Vehicle	Tg2576 UE2316	
BACE	0.266 ± 0.024	0.350 ± 0.052	0.289 ± 0.038	0.195 ± 0.028	.83
ADAM10	9.61 ± 2.51	8.98 ± 1.7	7.32 ± 1.01	5.06 ± 1.092	.47
PSD95	2.43 ± 0.595	2.84 ± 0.698	1.52 ± 0.37	2.04 ± 0.53	.16
Synaptophysin	195.2 ± 42.14	150.4 ± 0.397	133 ± 23.56	89.79 ± 15.39	.27
CD31	0.031 ± 0.007	0.042 ± 0.009	0.036 ± 0.009	0.019 ± 0.001	.77
Iba	0.144 ± 0.037	0.195 ± 0.043	0.238 ± 0.026	0.221 ± 0.032	.67
IDE	0.01 ± 0.0008	0.015 ± 0.002^a^	0.0096 ± 0.001	0.015 ± 0.003^a^	.005**

Data are mean ± SEM from Western blot densitometry, normalized to β-tubulin. A two-way ANOVA analysis was performed and the treatment effect of UE2316 is shown.

** signifies significance in statistical test.

a *P* < .04 for IDE for effect of UE2316 within each genotype by post hoc Fisher's least significant differences tests.

**Table 3.** Antibody Table

Peptide/Protein Target	Antigen Sequence (if Known)	Name of Antibody	Manufacturer, Catalog Number, and/or Name of Individual Providing the Antibody	Species Raised (Monoclonal Or Polyclonal)	Dilution Used
Aβ	Amino acids 1–16 of Aβ	6E10	Covance, Cambridge Bioscience, SIG-39300-200	Mouse monoclonal	1:1000
Iba1	C-TGPPAKKAISELP	Iba1	Abcam, ab5076	Goat polyclonal	1:1000
IDE	Amino acids 950 to c terminus	IDE	Abcam, ab32216	Rabbit polyclonal	1:1000
PSD-95	Amino acids 50–150 mouse PSD95	PSD95	Abcam, ab18258	Rabbit polyclonal	1:1000
ADAM10	PQRQRPRESYQMGHMRR	ADAM10	Abcam, ab1997	Rabbit polyclonal	1:1000
Synaptophysin		Synaptophysin	Abcam, ab7837	Rabbit polyclonal	1:1000
CD31	C terminus mouse CD31	CD31	Abcam, ab28364	Rabbit polyclonal	1:1000
BACE1	N terminus amino acids 46–62	BACE1	Sigma, B0681	Rabbit polyclonal	1:1000
β-Tubulin		β-Tubulin	Merck-Millipore, MAB3408	Mouse monoclonal	1:500

PSD95 and synaptophysin, markers of synaptic density (30, 31), were unaffected by UE2316 (Table 2). In addition, microglial density was not increased as evidenced by no change in Iba1 levels (32) (Table 2). CD31 staining was also conducted to probe for potential changes in cerebral vascular density because the 11β-HSD1 null mice exhibit enhanced angiogenesis (33), but no effect of UE2316 was observed (Table 2).

## Discussion

We have generated UE2316, which is a potent and selective inhibitor of 11β-HSD1 in the mouse brain. UE2316 administered either systemically or directly to the brain induces improvements in memory in cognitively impaired rodents. In aging mice the effects of UE2316 recapitulate those of other selective 11β-HSD1 inhibitors (15, 16) and provide further evidence that short term inhibition of 11β-HSD1 in the brain improves memory impairments associated with aging. Moreover, our data demonstrate that these improvements are associated with inhibition of 11β-HSD1 specifically in the brain since mice treated with icv administration with subsystemic doses display memory improvements comparable to those observed in mice treated systemically. The effects are evident across a range of behavioral tasks that involve the hippocampus, in contrast with the attenuation of contextual fear-associated memory that we previously reported with UE2316, which is likely mediated in different brain regions (19). It is likely that these short-term improvements in memory are due to the effects of reduced intracellular corticosterone in regions of the brain such as the hippocampus, which are sufficient to reverse the memory-impairing effects of glucocorticoids in aged mice (17). Structural changes to the hippocampus, such as synaptic and dendritic atrophy, may be reversed by reduced intracellular glucocorticoid levels over a period of hours and weeks, respectively, and could be responsible for the short-term memory improvements observed in these studies (34, 35). However, we also observed significant improvements in memory with long-term 11β-HSD1 inhibition, which may be mediated by structural hippocampal changes. It should be noted that behavioral testing was carried out only in male mice and the exploration of any potentially sexually dimorphic effects of 11β-HSD1 inhibition will require separate, comparative studies in male and female mice.

There is now substantial evidence from studies in rodents and in humans that reductions in 11β-HSD1 activity in the brain provides beneficial effects on the cognitive decline associated with aging (15, 16, 18, 23). However, to date no studies have been published that assess the effects on 11β-HSD1 inhibition in rodent models of AD. We found that short-term (29 d) treatment of already cognitively impaired 14-month-old Tg2576 mice with UE2316 led to improvements in memory during the passive avoidance task. UE2316 also improved latency in wild-type mice but to a lesser extent than in Tg2576 mice. Our data also demonstrate that long-term inhibition of 11β-HSD1 in Tg2576 mice maintains cognitive performance with aging because age-matched mice without UE2316 treatment are cognitively impaired in tests performed from 16 months of age onward. Moreover, cognitive improvement with 11β-HSD1 inhibition is maintained in the presence of significant Alzheimer's pathology.

In the Tg2576 mouse strain, Aβ plaques have been shown to develop from 9 months of age onward, associated with impairments in cognitive ability in memory tests from 10 to 12 months (25). As expected, when we examined the brains of the Tg2576 mice, Aβ plaques were observed in the cortex, amygdala, and hippocampus. Short-term UE2316 treatment substantially decreased the Aβ plaque number and the area in the cortex and amygdala of Tg2576 mice. In contrast, in a separate cohort of mice from which those with retinal impairment were excluded, we observed less Aβ plaque burden and no statistically significant effects of chronic UE2316 administration. This likely reflects the differences in animals with and without visual impairment. Overall, the results suggest that treatment with UE2316 has a disease-modifying effect on amyloid plaque deposition in Tg2576 mouse brains by impairing plaque accumulation. However, our findings dissociate cognitive improvement from Aβ plaque pathology after chronic treatment, suggesting the mechanism of improved cognition with 11β-HSD1 inhibition is not mediated solely through reduced plaque burden. Whatever the mechanism, the effect on cognition is likely associated with lowered intracellular glucocorticoid levels in the brain and the consequent altered balance of glucocorticoid and mineralocorticoid receptor action (23).

Additionally, we observed an increase in cortical IDE protein levels with short-term UE2316 treatment. This increase may explain, in some part, the reduction in plaque numbers and plaque area in the drug-treated mice because previous studies have demonstrated that overexpression of IDE or neprilysin in the neurons of transgenic mice significantly reduced brain Aβ levels and slowed or completely prevented amyloid plaque formation in APP TG mice (36), whereas IDE null mice have excess cerebral accumulation of Aβ (37). In humans, genome-wide association studies report a higher susceptibility to AD in Finnish patients with polymorphisms of IDE, suggesting that the rate of Aβ degradation may be an important factor in the development of human AD (38). However, the lack of IDE induction with chronic UE2316 administration, in the face of persisting benefits for cognitive function, suggests that other pathways are involved in cognitive protection with 11β-HSD1 inhibition. Alternatively, Tg2576 mice selected for intact vision may be relatively resistant to glucocorticoid effects on IDE and Aβ turnover.

These preclinical results support the concept that 11β-HSD1 inhibition may be efficacious for memory impairments not only with aging but also in AD. A recent phase 2 clinical trial in patients with mild-moderate AD was halted; however, when the selective 11β-HSD1 inhibitor ABT-384 failed to show noninferiority against donepezil for the primary end point of Alzheimer's Disease Assessment Scale-cognitive subscale (ADAS-Cog) score (39). Although a pharmacodynamic study of ABT-384 using stable isotope D4-cortisol tracer has been reported (40), it remains uncertain whether ABT-384 inhibited 11β-HSD1 adequately in brain because of the following: data were presented for only two control subjects without administration of ABT-384; after ABT-384 administration, D3-cortisol levels (generated by 11β-HSD1) (41) were very low in plasma, consistent with systemic enzyme inhibition, and this may account for the undetectable levels of D3-cortisol in CSF; and the maximum CSF concentrations of ABT-384 achieved, which were present for only a short time after dosing, were not high enough to inhibit 11β-HSD1 by more than 10%, according to the published potency of the compound. The benefits of 11β-HSD1 inhibition may be apparent only in the treatment of early disease, when the combination of symptomatic cognitive improvement and potential for disease modification we have observed in the mouse model of AD may be most useful.
